# Prognostic Significance of Preoperative Serum Carcinoembryonic Antigen Varies with Lymph Node Metastasis Status in Colorectal Cancer

**DOI:** 10.1155/2021/4487988

**Published:** 2021-12-27

**Authors:** Jing Jia, MinZhe Li, Wenhao Teng, Lin Wang, Weidong Zang, Jun Xiao, Ying Chen

**Affiliations:** ^1^Central Laboratory, Fujian Medical University Cancer Hospital& Fujian Cancer Hospital, Fuzhou, China; ^2^General Surgery Department, Beijing Chao-Yang Hospital, Capital Medical University, Beijing, China; ^3^Department of Gastrointestinal Surgery, Fujian Medical University Cancer Hospital& Fujian Cancer Hospital, Fuzhou, China

## Abstract

**Background:**

Preoperative serum level of carcinoembryonic antigen (pCEA) is generally recognized as a prognostic factor for colorectal cancer (CRC), but the stage-specific role of pCEA in colorectal cancer remains unclear.

**Objective:**

We investigated the prognostic significance of pCEA levels in different tumor stages of nonmetastatic CRC patients.

**Methods:**

Six hundred and fifteen CRC patients at stage I–III were retrospectively analyzed. All of them received curative tumor resection. The *X*-tile program was used to generate stage-specific cutoff values of pCEA for all patients and two subpopulations (lymph node-positive or -negative). The prognostic significance of pCEA was assessed using Kaplan–Meier analysis and Cox proportional hazards regression analysis. A nomogram model that combined pCEA score and clinical feature indexes was established and evaluated.

**Results:**

Two cutoff values were identified in the study population. At a cutoff value of 4.9 ng/mL, a significantly higher 5-year overall survival (OS) rate (82.16%) was observed in the pCEA-low group (<4.9 ng/mL) compared with 65.52% in the pCEA-high group (≥4.9 ng/mL). Furthermore, at the second cutoff value of 27.2 ng/mL, 5-year OS was found to be only 40.9%. Stratification analysis revealed that preoperative serum level of pCEA was an independent prognostic factor (OR = 1.991, *P* < 0.01) in the subpopulation of lymph node metastasis (stage III) patients, and the relative survival rates in the pCEA-low (≤4.9 ng/mL), pCEA-medium (4.9–27.2 ng/mL), and pCEA-high (≥27.2 ng/mL) groups were 73.4%, 60.5%, and 24.8%, respectively (*P* < 0.05). However, no such effect was observed in the lymph node nonmetastasis (stage I and II) subgroup. The established nomogram showed acceptable predictive power of the 5-year OS rate (C-index: 0.612) in lymph node-positive CRC patients, with an area under the curve value of 0.772, as assessed by ROC curve analysis.

**Conclusions:**

Pretreatment serum CEA levels had different prognostic significance based on the lymph node metastasis status. Among stage III CRC patients, pCEA was an independent prognostic factor. Five-year OS rates could be predicted according to the individual pCEA level at the different cutoff values.

## 1. Introduction

Carcinoembryonic antigen (CEA) is a tumor marker in gastrointestinal cancers, particularly colorectal cancer (CRC). It belongs to a superfamily of glycoproteins expressed on cell membranes that play an important role in cell recognition and adhesion. Since it was first described in 1965 [1, 2], the biological function and clinical significance of CEA in CRC have been intensively investigated [3–5].

It is generally accepted that preoperative CEA (pCEA) is not suitable for screening or diagnostic purposes in CRC [6]. However, the prognostic role of pCEA in CRC was suggested by many studies. Based on these findings, two important issues were published in 2000. First, the American Joint Committee on Cancer proposed that pCEA can be added to the TNM staging system as an additional stratification factor for CRC on the basis of the presence or absence of a preoperative serum level of pCEA ≥5 ng/mL [7]. Second, the College of American Pathologists Expert Groups included pCEA concentration as a category I prognostic marker for CRC [8]. Six years later, two international independent organizations, the American Society of Clinical Oncology and the European Group on Tumor Markers, both recommended pCEA as a prognostic indicator in CRC [9, 10].

Despite a consensus being reached on the role of pCEA in CRC, the practical application of pCEA as a routine tool was still obstructed by many uncertainties. One was the definition of an “elevated” pCEA level. Because the elevation of preoperative pCEA was associated with the development of tumor stage, the traditional use of a single pCEA cutoff value for prognostic assessment may not be suitable for all CRC patients at other stages [11–13]. Many recent studies suggested the significance of using different cutoff values for prognostic evaluation [12, 14, 15]. On the basis of this hypothesis, we performed an evaluation of different cutoff values of pCEA in subgroups of CRC patients with or without lymph node metastasis, hoping to further develop the application value of this traditional tumor marker in clinical practice.

## 2. Materials and Methods

### 2.1. Patients

Six hundred and fifteen eligible patients who were diagnosed with nonmetastatic resectable CRC during the period January 2010 to December 2013 at Fujian Cancer Hospital were recruited. All patients underwent surgery. Data for preoperative serum CEA level, clinicopathological features, and individual characteristics from each patient were retrospectively retrieved from the patient records within the hospital database.

In keeping with the 8^th^ edition of the TNM classification system [16], the inclusion criteria included the following: (1) pT_X_N_X_M0 resectable CRC; (2) adenocarcinoma confirmed by histopathological examination; (3) physical fitness suitable for surgery; and (4) without receiving any type of adjunctive therapy. The exclusion criteria included the following: (1) over 90 years old; (2) with preexisting or other concomitant cancers; (3) distant metastatic diseases; (4) noncurative resection; (5) multiple primary malignancies; (6) died within 30 days after surgery.

All patients were followed up by letter or telephone interview. The last follow-up was conducted in January 2018, and the median follow-up period was 57 months (range, 7–90 months). (1) Over 85 years of age (*n* = 21); (2) with previous or other concomitant cancers (*n* = 11); (3) distant metastatic disease (*n* = 31); (4) noncurative resection (*n* = 12); (5) multiple primary malignancies (*n* = 11); (6) mortality within 30 d after surgery (*n* = 0). The process diagram of this article is as Line 1.

### 2.2. Statistical Analysis

Statistical analyses were conducted using SPSS software version 19.0. The distributions of baseline characteristics were compared using either unpaired *t*-test or ANOVA test. The cutoff values of pCEA were determined and analyzed using the *R* (survival ROC) and *X*-tile program, which identified the cutoff with the minimum *P* value from log-rank *χ*^*2*^ statistics for the categorical pCEA in terms of survival. Meaningful factors were calculated using the logistic regression method extracted for further analyses. The overall cumulative probability of survival was calculated using the Kaplan–Meier method, and differences were evaluated using the log-rank test.

To evaluate the numerical weight of each factor, particularly the influence of pCEA, for predicting the long-term clinical outcome, a nomogram model integrating pCEA level and the AJCC 8th staging system were developed using *R* 4.0 software. The concordance index (C-index), receiver operating characteristic (ROC) curve, and internal calibration plot were further used to evaluate predictive performance. *P* values of <0.05 were considered statistically significant.

## 3. Results

### 3.1. Clinicopathological Characteristics of Study Subjects

Among 615 CRC patients, there were 87 (14.15%) at stage I, 237 (38.54%) at stage II, and 291 (47.32%) at stage III. The 1-, 3-, and 5-year overall survival (OS) rates were 99.35%, 97.56%, and 74.30%, respectively. The distribution of pCEA level ranged from 0.3 to 266.2 ng/mL. Detailed information of recruited patients is summarized in Table 1.

### 3.2. Prognostic Significance of pCEA in Nonmetastatic CRC Patients

**Figure 1:**
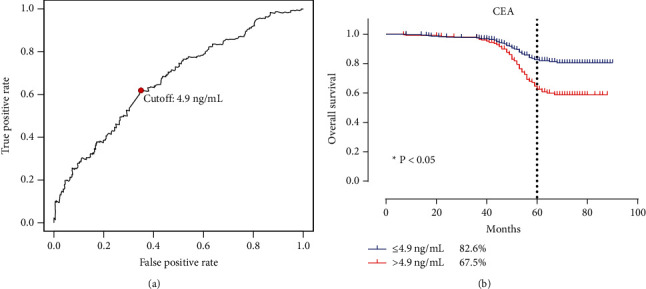
Survival analysis of CRC patients. (a) The best cutoff value calculated by R (survival ROC). (b) The 5-year survival rate of two subgroups. ROC: receiver operating characteristic curve.

The prognostic significance of CEA was analyzed according to the *X*-tile program. Two cutoff values were detected. At the first cutoff value of 4.9 ng/mL, a significantly higher 5-year OS rate (82.16%) was observed in the CEA-low group (<4.9 ng/mL) as compared with 65.52% in the CEA-high group (≥4.9 ng/mL) (*P* < 0.05) (Figure 1).

**Figure 2:**
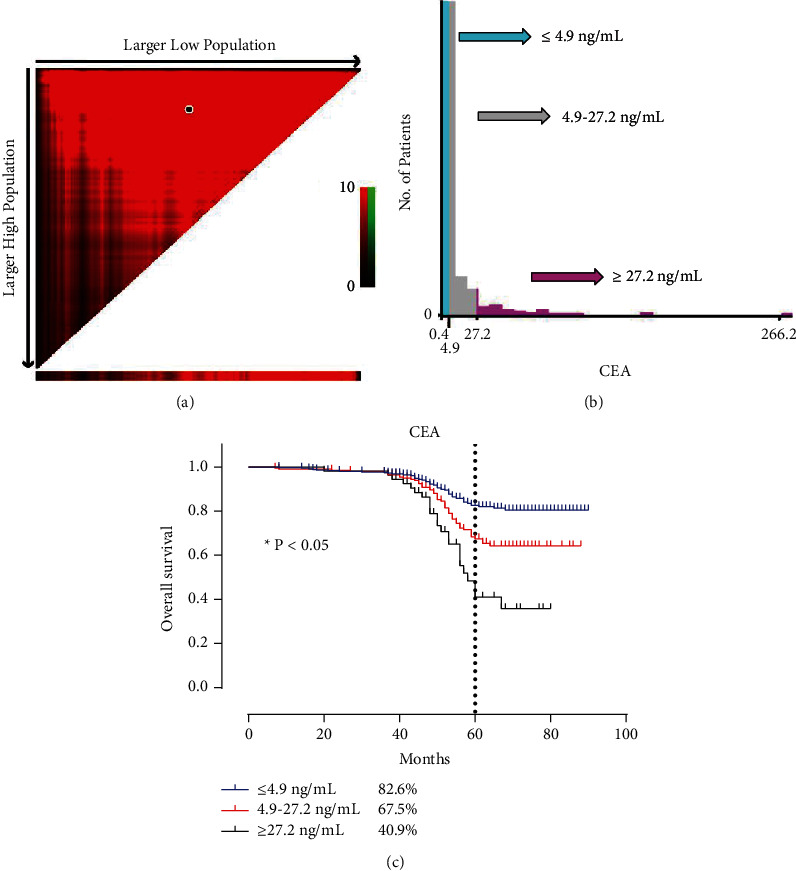
Determination of optimal cutoff values of further distinguishing high-risk groups of CRC patients. (a, b) Identification of the optimal cutoff value of pCEA by X-tile. (c) Survival analysis for low pCEA (less than 4.9), medium pCEA (4.9 to 27.2), and high pCEA (more than 27.2) groups. CEA: carcinoembryonic antigen.

At the second cutoff value of 27.2 ng/mL, a significantly worse prognosis population was identified in patients with CEA levels more than 27.2 ng/mL, with a 5-year OS rate of only 40.9%. Thus, our data could stratify nonmetastatic CRC patients with different survival risks, i.e., CEA-low (<4.9 ng/mL), CEA-medium (4.9–27.2 ng/mL), and CEA-high (>27.2 ng/mL) groups. The relative 5-year survival rates of these three subgroups were 82.6%, 67.5%, and 40.9%, respectively (*P* < 0.05**)** (Figure 2).

Multivariate analyses revealed *T*_4_ stage, *N*_2_ stage, and CEA level were independent prognostic factors for nonmetastatic CRC (Table 2).

### 3.3. Prognostic Significance of pCEA in Subpopulations with Different Lymph Node Metastasis Statuses

**Figure 3:**
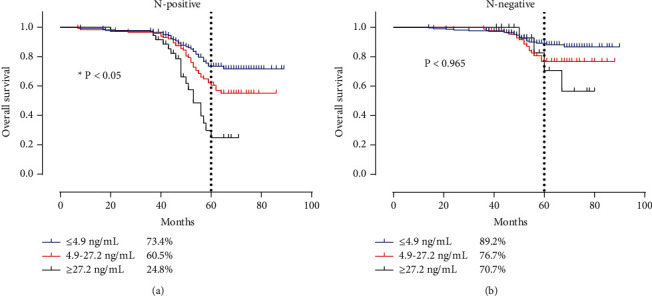
Survival curve of CRC patients according to different statuses of lymph node metastasis. (a) Lymph node metastasis group. (b) None lymph node metastasis group.

To address the question of whether there was a difference between subgroups of patients with or without lymph node metastasis regarding the prognostic performance of pCEA, survival analyses were further conducted using the created cutoff values. In the subgroup of lymph node metastasis (pN1–2), the 5-year survival rates of the three subgroups, pCEA-low (≤4.9 ng/mL), pCEA-medium (4.9–27.2 ng/mL), and pCEA-high (≥27.2 ng/mL), were significantly different at 73.4%, 60.5%, and 24.8%, respectively (*P* < 0.01) (Figure 3(a)). However, no significant differences were observed in the lymph node nonmetastatic (pN0) subgroup, at 89.2%, 76.7%, and 70.7%, respectively (*P* > 0.05) (Figure 3(b)).

Univariant analysis further confirmed that gender (OR = 1.789), pCEA (OR = 1.875), and T4 category (OR = 2.555) were independent factors for lymph node metastasis patients. Meanwhile, pCEA (OR = 1.610) was independent factor for patients with negative lymph nodes (Table 3). Multivariate analysis further confirmed that pCEA (OR = 1.991) and T4 category (OR = 2.101) were independent factors for lymph node metastasis patients (Table 4).

### 3.4. Establishment of a Predictive Nomogram

**Figure 4:**
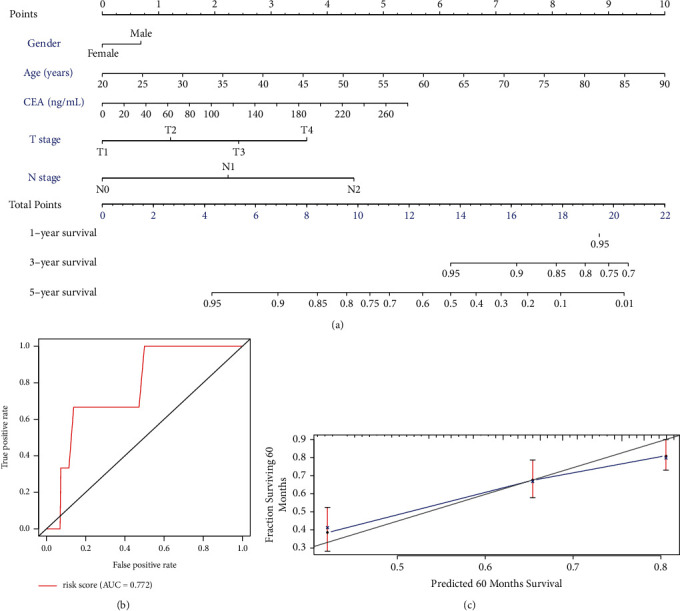
Integration model for predicting the 5-year survival probability using the nomogram. (a) The 5-year probability of death for a patient is located on the total points axis (bottom) by summing up the total points assigned to each variable at the scales shown above, as indicated with the lines drawn downward to each axis. (b) The result of ROC curves. (c) The calibration curve for the new nomogram model for 5 years. Gray: ideal model; blue: the new nomogram model. CEA: carcinoembryonic antigen; ROC: receiver operating characteristic curve.

Because pCEA level was an independent prognostic factor in stage III CRC patients, we constructed a prognostic nomogram that included gender, age, pCEA level, *T* category, and *N* category to predict 3- and 5-year OS in this subgroup (Figure 4(a)). The total points obtained from this model by summing the points of each variable could be used to estimate the 3- and 5-year OS rates of each patient. When the pCEA level was in the range of 5.0 to 27.2 ng/mL and pCEA was higher than 27.2 ng/mL, its contribution to the prognosis of the disease was similar to N_1_ and N_2_ stage, respectively.

The internally validated Harrell's C-index was 0.612, while the area under the curve value in predicting 5-year OS reached 0.772 for this model (Figure 4(b)). Furthermore, the calibration plot analysis showed that the 5-year survival probability predicted by the nomogram model had optimal agreement with the actual observation (Figure 4(c)).

## 4. Discussion

Considering that high preoperative CEA levels were closely correlated with tumor load, pCEA is generally identified as a biomarker for indicating multiple cancers [17]. However, the clinical prognostic value of pCEA in CRC remains unclear. There have been many discussions in previous studies on how to practically use pCEA level as a prognostic factor in CRC cases and whether the lymph node metastasis status will affect the significance of pCEA.

To address this, the current study collected 615 CRC cases based on similar endpoints. According to the CRC patients' survival results, pCEA level was found to be an independent prognostic factor for nonmetastatic CRC patients. Two cutoff values were detected in this study, accompanied by a significant prognostic outcome. The first cutoff was 4.9 ng/mL, which is similar to the conventional level of 5.0 ng/mL as used previously. The second cutoff was 27.2 ng/mL, which indicated a much worse 5-year OS. These findings are in line with the view of Park et al. that preoperative CEA levels could be used as a stratification parameter for identifying subsets of CRC patients with different prognostic outcomes [18].

Previous studies are unclear about the common effect of CEA and lymph node metastasis in predicting prognosis [19–21]. This problem may be related to the fact that subgroup analyses were not performed in these studies or that they used single cutoff values for pCEA. As the TNM stage of CRC increases, its tumor burden becomes heavier, which may also enable CEA to more accurately reflect the actual condition of the patient [22]. However, at earlier stages of clinical cases, there are many confounding factors, and thus pCEA may not be able to accurately assess tumor burden [23]. Therefore, this study divided CRC cases into subgroups according to the status of lymph node metastasis. Our results revealed that in patients with positive lymph nodes, pCEA level was an independent prognostic factor (OR = 1.991, *P* < 0.01). However, in the subgroup with negative lymph nodes, CEA level was not an independent prognostic factor (*P*=0.326). This result implied that the prognostic significance of CEA in CRC differs from TNM stage. Although our data suggested that pCEA level might be closely related to lymph node metastasis, consistent with previous research [24], the underlying mechanism remains unclear.

On this topic, some previous literatures also showed a similar research endpoint [25–27]. However, the number of samples included in the research of each literature is small, and the cutoff values obtained by statistical methods are not the same thing [28, 29]. Therefore, the research conclusions may be biased. The direction of this study is the clinical value of CEA in predicting metastatic CRC. Clinically, there are plenty of accumulated 615 CRC cases, with clear goals, strong pertinence, and reproducibility, which has clinical practical value and translational application value. In addition, most of the literatures directly use the upper limit of the normal reference value of 5.0 as the cutoff value, instead of being analyzed from the critical value data. This study combined CEA evaluation parameters of the clinical prognosis to calculate the cutoff point (4.9 ng/mL), which is different from the cutoff point setting in the previous literature, and the data are more reliable.

Interestingly, as shown by the nomogram model established in this study, we found that when pCEA was in the range of 5.0 to 27.2 ng/mL, its contribution to 5-year OS was equivalent to N_1_ staging. Moreover, a similar association was observed between pCEA levels higher than 27.2 ng/mL and *N*_2_ staging. These results are consistent with clinical observations and further support the idea that pCEA level is closely related to the status of lymph node metastasis. Therefore, through this analysis, it may be possible to initially explain the differences in previous clinical research.

Despite the current study proving the significant role of pCEA in CRC prognosis, there are some limitations. First, the inherent bias of patient selection could not be completely avoided, which could partly affect surgical outcomes. Second, the case number of patients with high serum CEA levels was relatively limited in this study, which reduced the statistical power. Third, the data collection was localized in one hospital, which suggests that the current results might not be applicable to different regions of China.

In conclusion, we demonstrated that pCEA levels can effectively predict prognosis in CRC patients with positive lymph node metastasis. When pCEA levels are higher than 4.9 ng/mL, they generally indicate worse and unfavorable tumor behavior and poor prognosis.

## Figures and Tables

**Table 1:** Demographic data of the 615 patients with CRC, *n* (%).

Characteristics	(n = 615)	%	pCEA level (ng/mL), median (min to max)
*Age (years)*	58.00 (20–87)		3.85 (0–266.2)

*Gender*			
Female	229	37.24	3.48 (0.3–266.2)
Male	386	62.76	4.01 (0.5–224.7)
*Location*			
Right	117	19.02	3.96 (0.6–77.25)
Left	151	24.55	3.34 (0.43–181.1)
Rectum	347	56.42	4.2 (0.3–266.2)

*TNM stage*			
I Stage	87	14.15	2.49 (0.5–32.2)
II stage	237	38.54	3.36 (0.3–224.7)
III stage	291	47.32	5.0 (0.43–266.2)

*N category*			
*N*_0_ category	324	52.68	3.0 (0.3–224.7)
*N*_1-2_ category	291	47.32	5.0 (0.43–266.2)

CEA: carcinoembryonic antigen.

**Table 2:** Multivariate analysis for CRC patients.

	*P* value	OR	95% CI used for Exp (B)	
			Lower	Upper
*Gender*	0.447	0.868	0.603	1.250
*Age*	0.234	1.031	0.747	1.516

*T category*				
*T*_1_	1 (reference)			
*T*_2_	0.107	1.350	0.937	1.945
*T*_3_	0.167	0.365	0.087	1.523
*T*_4_	0.000	1.902	1.686	3.014

*N category*				
*N*_0_	1 (reference)			
*N*_1_	0.650	0.630	0.085	4.643
*N*_2_	0.000	2.533	1.913	3.775

*Location*				
Right	1 (reference)			
Left	0.514	1.738	0.816	2.707
Rectum	0.442	1.262	0.698	2.282
*pCEA level*	0.000	1.931	1.396	2.421

CEA: carcinoembryonic antigen; OR: odds ratio; 95% CI: 95% confidence interval.

**Table 3:** Univariant analysis for CRC patients according to lymph node status.

	*N*+		95% CI used for Exp (B)		N0		95% CI used for Exp (B)	
	*P* value	OR	Lower	Upper	*P* value	OR	Lower	Upper
Gender	0.030^*∗*^	1.789	1.570	3.123	0.466	0.858	0.569	1.294
Age	0.201	1.034	0.818	1.052	0.289	1.055	0.814	1.088
*T*_4_ category	0.000^*∗*^	2.555	2.266	3.157	0.294	0.801	0.530	1.212
pCEA level	0.000^*∗*^	1.875	1.407	2.499	0.035^*∗*^	1.610	1.034	2.506

CEA: carcinoembryonic antigen; OR: odds ratio; 95% CI: 95% confidence interval; ^*∗*^the difference is statistically significant.

**Table 4:** Multivariant analysis for CRC patients according to lymph node status.

	*N*+		95% CI used for Exp (B)		N0		95% CI used for Exp (B)	
	*P* value	OR	Lower	Upper	*P* value	OR	Lower	Upper
Gender	0.161	0.735	0.478	1.130	0.614	1.200	0.591	2.438
Age	0.991	1.030	0.913	1.048	0.701	1.055	0.923	1.089
*T*_4_ category	0.000^*∗*^	2.101	1.736	3.321	0.453	0.559	0.122	2.558
pCEA level	0.000^*∗*^	1.991	1.471	2.742	0.326	1.005	0.995	1.015

CEA: carcinoembryonic antigen; OR: odds ratio; 95% CI: 95% confidence interval; ^*∗*^the difference is statistically significant.

## Data Availability

The data of this paper come from the local database of Fujian Medical University Cancer Hospital and Fujian Cancer Hospital.
